# Cerebral venous sinus thrombosis in HIV-infected patients: report of 2 cases

**DOI:** 10.11604/pamj.2013.16.4.3252

**Published:** 2013-09-04

**Authors:** Julius Chacha Mwita, Kgomotso Baliki, Ludo Tema

**Affiliations:** 1 Department of Internal Medicine, University of Botswana, Gaborone, Botswana; 2 Department of Internal Medicine, Princess Marina Hospital, Gaborone, Botswana

**Keywords:** Cerebral venous sinus thrombosis, HIV, cerebrovascular diseases, stroke

## Abstract

Infection with the human immunodeficiency virus (HIV) is associated with increased risk of cerebrovascular disease; however Cerebral Venous Sinus Thrombosis (CVST) is rarely associated with HIV-related cerebrovascular events. We describe two cases of HIV-positive patients who, at the same time, presented to our hospital with deep cerebral venous thrombosis and stroke.

## Introduction

Cerebral venous sinus thrombosis is a rare cause of stroke that often affects young adults and children [1]. About 75% of the affected adults are women, and direct causes or predisposing risk factors can be identified in up to 85% of patients [1]. They include local trauma and infection, prothrombotic states like nephrotic syndrome, anti-thrombin III deficiency, pregnancy, malignancy and the use of oral contraceptives[1–3]. Although venous thrombotic events (VTEs) are frequent among HIV patients, few cases of CVST have been reported[4, 5]. The mechanism of HIV-related thrombosis is complex and involves the intersection of HIV infection, highly active antiretroviral therapy (HAART) and traditional prothrombotic factors [1, 5, 6]. We report two HIV-positive females who presented with stroke secondary to cerebral venous sinus thrombosis. They were both on antiretroviral therapy that constituted zidovudine, lamivudine and nevirapine.

## Patients and observations

### Case report 1

A 17-year-old female presented with new onset right sided focal seizures that started on the day of presentation. She had three episodes prior to being seen at our casualty, each lasting about 5-10 minutes and was associated with confusion. At the casualty, she had a right sided tonic-clonic seizure that involved the face, upper and lower limbs. Four days prior to presentation, the patient started having a global headache that was associated with photophobia and painful eyes. She denied any fever, nausea, vomiting or a recent head trauma. Two weeks before, she had a tympanoplasty on the right ear for chronic suppurative otitis media without any immediate post-procedure complications. Her past medical history revealed a left ear tympanoplasty in 2011. She is HIV positive, acquired from her mother, and she has been on a combination of zidovudine, lamivudine and nevirapine since the age of 5 years. Her last CD4 cell count was 198cell/µL. Her mother died of HIV/AIDS when she was about 4 years old leaving her alone with her busy father. This has made her vulnerable to sexual abuses and she has had about eight reported episodes of rape since the age of 12 years. As a result, she was put on oral contraceptives (OCPs) since the age 13 years and was switched to injectable contraceptive Depo-Provera 4 months ago. She denied any history of alcohol intake, cigarette smoking or using illicit drugs. There was no family history of strokes, sudden deaths or clotting disorders.

Examination after convulsion revealed an afebrile and anxious patient with a regular pulse rate of 127 beats/minute, a respiratory rate of 28/minute and a blood pressure of 131/43 mmHg. She had no lymphadenopathy and there were no bleeding or discharge from both ears. She had nuchal rigidity but without any neurological deficits. Examination findings of the abdomen, cardiovascular and respiratory systems were unremarkable. The patient's initial investigations revealed normal full blood count, renal and liver function tests. Her random blood glucose was 4.9mmol/L and she had negative antinuclear antibody test results. Cerebrovascular fluid examination revealed a slightly turbid fluid with 6 white cells/mm^3^ and 75 red blood cells/mm^3^. A computed tomography of the brain with contrast showed diffuse meningeal enhancement, white matter oedema of the left brain hemisphere and a filling defect at the confluence of the transverse cerebral veins suggestive of thrombosis of the sagittal vein. The patient was admitted for anticonvulsants and anticoagulation on the wards.

**Figure 1:**
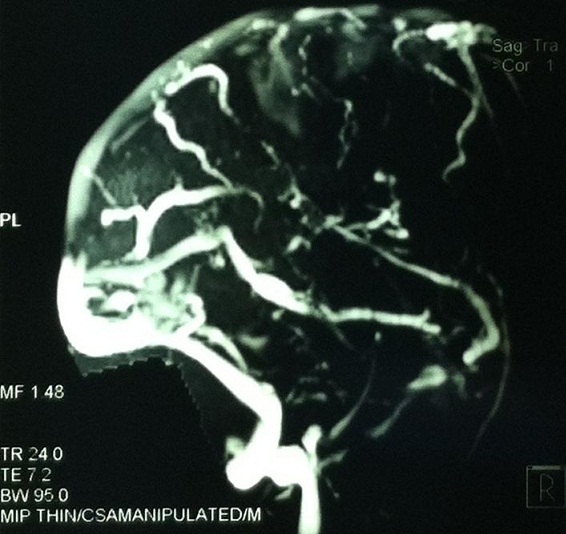
Magnetic Resonance Venography demonstrating thrombosis of the superior sagittal sinus

One day after admission she had about 10 episodes of sided convulsions, with residual right sided hemiparesis. The power of all the muscle groups in the right upper and lower limbs was grade 2/5. A Magnetic Resonance venography established superior sagittal and left transverse sinuses thrombosis (Figure 1). Furthermore, there were venous infarction with haemorrhagic component and oedema in the left frontal, temporal, occipital and parietal lobes. In addition to continuation of anticoagulation, anticonvulsants were optimized to control convulsions. There was a reduction of seizure episodes over the subsequent five days and the patient's neurological deficit slowly normalized inthe next three weeks. She was then discharged home with mild hemiparesis and her subsequent outpatient visits showed no neurologic deterioration.

### Case report 2

A 44-year old female presented with a constant, throbbing and worsening headache over the past three days. The headache was generalized in nature, associated vomiting, photophobia, dizziness and diplopia. There was no history convulsion, limb weakness or parasthesia. She denied any history of trauma, fever or any loss of consciousness. Her past medical history was significant of HIV disease for the past 10 years. She was on Zidovudine, Lamivudine and Nevirapine treatment, and her last CD4 count was 293 cell/µl two months ago. She did not smoke cigarettes and had never used contraceptive pills. There was no family history of strokes, sudden deaths or clotting disorders. On examination, the patient was teary, in obvious discomfort, afebrile and with normal vital signs. Her Glasgow coma scale was GCS 15/15 with normal pupils and extra-ocular muscle movement. There were signs of meningism without any neurological deficits. Examinations of other systems were unremarkable and she had normal baseline blood tests. Lumbar puncture had an opening pressure of 23 cm H2O with a closing pressure of 10 cmH2O and normal laboratory cerebrospinal fluid results. The patient was admitted for observation in the ward and discharged three days later when her CSF culture results were negative. Two days after discharge, she was readmitted with worsening headache, photophobia and diplopia. On examination she had normal vital signs, neck stiffness and bilateral lateral rectus muscle weakness. A repeat lumbar puncture showed opening and closing pressures of 30cm and 15cm of H_2_O respectively. The headache improved after lumbar puncture, and her cerebrospinal fluid microscopy and culture results were once more negative. A non-contrast computed tomography showed ill-defined sulci and hyper-density along the sagittal, right transverse and sigmoid sinuses. With intravenous contrast, filling defects were noted in the above sinuses. There were no obvious haemorrhages, infarcts or mass effect noted. We made a diagnosis of thrombosis of the superior sagittal, right transverse and left sigmoid sinuses. The patient was started on low molecular weight heparin and subsequently continued on oral warfarin. Few days later, she had five episodes of generalised seizures without any residual neurological deficits. Fundoscopy revealed papilloedema that subsequently improved with intravenous Manitol. Similarly, her seizures were controlled by phenytoin and she was discharged about three weeks later without any neurologic deficit.

## Discussion

Cerebral venous sinus thrombosis is a rare condition that often presents with thrombosis in the cerebral venous or dural sinuses, and rarely in the cortical (superficial) veins [2]. As in our patients, the superior sagittal and left transverse sinuses are the most affected sinuses [7]. CVST affects young adults and children, and represents about 1% of all strokes[1, 2]. It occurs in about 3-4 cases per million population, mainly in women due to the use of oral contraceptive pills and the postpartum state[1]. There are several factors associated with CVST, and it is not uncommon to find multiple factors in a single patient [1]. Aetiological factors include hypercoagulable states, inflammatory and infectious diseases such as facial infections, dental abscesses, otitis media, mastoiditis, endocarditis and septicaemia [1, 2]. The present cases occurred in female patients, and one of them had a recent tympanoplasty due to chronic otitis media. Although venous thrombotic events (VTEs) are frequent among HIV patients, few cases of CVST have been reported [4, 5]. Mechanisms for the observed hypercoagulability in HIV infected patients are multifactorial and include the presence of antiphospholipid antibodies and deficiency of natural anticoagulants such as protein C, protein S, heparin cofactor II, and antithrombin [5]. Some studies have reported a high prevalence of antibodies against protein S among HIV infected patients, leading to significantly low protein S activity in about 31%-76% of patients [8]. Although protein S deficiency is not correlated with HIV disease severity it appears that thrombosis is highly correlated with low CD4 counts (^3^), the presence of opportunistic infections, malignancies, or autoimmune disorders [9, 10]. Our patients had no opportunistic infections and only one of them had CD4 count of less than 200/mm^3^. Nevertheless, we could not determine their protein C and protein S levels. While one patient had a prothrombotic state because of contraceptives use and otitis media, the second patient's thrombosis risk could only be attributed to her HIV seropositivity. Both patients used HIV antiretroviral regimen that contained Zidovudine, Lamivudine and Nevirapine. Although the absolute risk of thrombosis in patients not using combination antiretroviral therapy is about 6-fold in comparison with a healthy population of comparable age, there is additional risk of thrombosis in patients on HAART [6, 10, 11]. The prothrombotic effect of HAART is nevertheless more pronounced when a combination that includes protease inhibitors (PIs) is used [6, 12]. Protease inhibitors based therapy promotes thrombosis by inducing platelets and endothelial dysfunction [12]. It is however remains unclear whether non-protease inhibitors are prothombotic [11].

The clinical presentations of CVST are quite variable and result from mass effect of the enlarging thrombus as well as the consequential increased intracranial pressure[1]. As a result, swollen veins, oedema haemorrhages and infarction are typically found on imaging[1]. Headache is the commonest symptom, present in 95% of CVST patients [13]. Seizures occur in about 47%, and may be focal in about half of the patients [1, 13]. Other reported symptoms include hemiparesis, aphasia, coma and papilloedema [1, 13]. Our patients presented with headache, seizures, hemiparesis and papiloedema. The diagnosis of CVST is usually done by the Computerized tomographic (CT) and magnetic resonance imaging (MRI) scans[1]. However, magnetic resonance venography is preferred as it outlines the occluded sinuses as well as associated cerebral oedema and venous infarctions [14]. Initial management involves patient's stabilization and prevention or reversing cerebral herniation by the use of mannitol or surgery[1]. Despite the risk of haemorrhage, anticoagulation is advised to stop the propagation of thrombosis and prevent pulmonary thrombosis [1]. Even in patients with evidences of haemorrhagic brain infarction, anticoagulation has safely been used [1, 7]. The prognosis of CVST is generally good, with more than 80% of all patients having full neurologic recovery [1]. Our patients were given lower molecular weight heparin for five days and subsequently continued on warfarin. They both had remarkable clinical improvement, and were discharged on warfarin that they will use for at least 6 months with a target international normalized ratio of 2.5. The duration of oral anticoagulation is often six months after a first episode of sinus thrombosis, or longer in the presence of predisposing factors[1]. Although there are limited evidences, local thrombolysis into the sinus may be attempted in patients with sinus thrombosis who deteriorate despite of anticoagulation [15]. Increased intracranial pressure can be managed by the treatment with acetazolamide, repeated lumbar punctures or surgical drainage of the cerebrospinal fluid if symptoms persist[1].

## Conclusion

Human immunodeficiency virus infection is associated with increased thrombosis. However, mainly episodes of deep venous thrombosis (DVT) and pulmonary embolism are often reported. Sinus thrombosis is fairly common and should be considered as a differential in HIV patients with persistent neurologic symptoms or signs despite of normal CSF findings. Although the clinical diagnosis of CVDT is a challenge, its treatment is often rewarding for most patients.
